# A Brief Updated Review of Advances to Enhance Resveratrol’s Bioavailability

**DOI:** 10.3390/molecules26144367

**Published:** 2021-07-20

**Authors:** Konrad de Vries, Morné Strydom, Vanessa Steenkamp

**Affiliations:** Department of Pharmacology, Faculty of Health Sciences, University of Pretoria, Private Bag x 323, Arcadia 0007, South Africa; k-de-vries@hotmail.com (K.d.V.); morne.strydom@up.ac.za (M.S.)

**Keywords:** resveratrol, in vitro studies, in vivo studies, bioavailability, analogues, synergism

## Abstract

Resveratrol (RES) has a low bioavailability. This limitation was addressed in an earlier review and several recommendations were offered. A literature search was conducted in order to determine the extent of the research that was conducted in line with these recommendations, along with new developments in this field. Most of the identified studies were pre-clinical and confirmed the heightened activity of RES analogues compared to their parent compound. Although this has provided additional scientific kudos for these compounds and has strengthened their potential to be developed into phytopharmaceutical products, clinical trials designed to confirm this increased activity remain lacking and are warranted.

## 1. Introduction

The health benefits of resveratrol (RES) have been widely published. To this end, 244 clinical trials had been completed with a further 27 ongoing at the end of 2019. Clinical data from these studies were reviewed by Singh et al. [1], who referred to the rapid metabolism and poor bioavailability of RES which limits its therapeutic use. The low RES bioavailability and potential ways in which this may be overcome was made the focus of a previous review by the current authors [2]. Several recommendations were made, one being the use of analogues which have increased bioavailability.

Since 2017, a number of pre-clinical studies focused on RES analogues have been published. Methoxylated analogues (most noteworthy, tetramethoxystilbene) showed anticancer activity by reducing MCF-7 breast cancer cell viability and inducing cell cycle arrest *in vitro*. These effects were superior to the parent compound, RES [3]. Another RES analogue, isorhapontigenin (ISO), was reported to inhibit human bladder cancer stem cell-like phenotypes in vitro [4]. ISO also protected against doxorubicin-induced cardiotoxicity in an animal model [5]. Resveratrol trimethyl ether (trans-3,5,4′-trimethoxystilbene, RTE) was shown to offer protection against atherosclerosis by suppressing plaque formation in the aortas of apolipoprotein E deficient mice by reducing macrophages and cholesterol levels [6].

Efficacious vaccines and treatments for COVID-19 are currently a research priority. Publications of RES for this role have emerged. Ter Ellen et al. [7] demonstrated that RES and its analogue, pterostilbene, exert antiviral effects against SARS-CoV-2 in a dose-dependent manner, possibly by inhibiting the viral replication cycle in vitro. RES was also shown to play a crucial role in the major pathophysiology pathways involved in SARS-CoV-2 infections by regulating the renin-angiotensin system and expression of angiotensin-converting enzyme 2, immune system modulation, and downregulation of pro-inflammatory cytokines [8]. These may therefore be promising antiviral agents against SARS-CoV-2 and should be investigated further in clinical trials. A novel administration of an RES and zinc combination using nano-carrier delivery systems, or a pterostilbene-zinc combination without a nano-carrier system, have both been suggested as possible mono and adjunct therapies for mild COVID-19 [9]. 

The purpose of this review is to update current information and to highlight the advances in identifying RES lead compounds that have the potential to be developed further.

## 2. Results and Discussion

### 2.1. Synergism of RES with Other Phytochemicals 

Although the authors of the previous review recommended that further research be conducted on the co-supplementation of RES with piperine, there was no new information on this combination. Conversely, results of the combination of piperine with oxyresveratol (an RES analogue) were available.

The biflavone ginkgetin, isolated from leaves of Ginkgo biloba, has been reported to contain a number of pharmacological activities including inhibition of different cancers [10,11,12,13]. When this compound was combined with RES, a synergistic effect was noted which resulted in the suppression of the angiogenic properties of the vascular endothelial growth factor (VEGF) [14]. This anti-angiogenesis activity was evident in in vitro cell proliferation, cell migration, and tube formation assays, as well as in in vivo studies in colon cancer xenograft mice models [14]. The vascular density of these tumors reportedly decreased by ~38% in mice receiving the combination treatment. Regarding the inflammatory response, the combination significantly reduced TNF-α and IL-6 cytokines by 85% and 66%, respectively. This synergistic activity was ascribed to the two compounds binding to different sites on VEGF [14].

### 2.2. Prodrugs

A group of researchers investigated the efficacy of 3,5,4′-tri-O-acetylresveratrol (TARES) in an animal model of acute respiratory distress syndrome [15]. TARES pre-treatment inhibited pulmonary inflammation and oxidative stress in seawater-induced lung injury in vivo [15]. TARES may prove to be an effective prodrug, capable of increasing the bio-efficacy of RES by elevating plasma levels of free trans-resveratrol. Further investigation, such as pre-clinical safety evaluation is required to establish whether or not TARES could be used in a clinical setting.

The alkylated derivatives of RES are prodrugs that have also shown promise for potential clinical use. These compounds were evaluated for toxicity as well as for their neuroprotective ability in a zebrafish in vitro model [16]. The most promising piceid acylated prodrug, [resveratrol-3-O-(60-O-octanoyl)-b-D-glucopyranoside], was then subjected to further investigation in a pre-clinical model of Huntington’s disease (HD) to determine its efficacy in treating neurodegeneration [16]. In this experiment, acetylcholinesterase (AChE) activity of zebrafish embryos that had been challenged with pentylentetrazole (a competitive GABA antagonist), before and after treatment with the prodrugs, was determined. Recovery of AChE activity after treatment with the parent compound RES was 92%, whereas 100% of AChE activity was noted when treated with the prodrug [16]. In a preclinical model of HD, both the prodrug and RES improved locomotor activity and prevented weight loss in mice to a similar degree [16]. The therapeutic potential of resveratrol-3-O-(60-O-octanoyl)-b-D-glucopyranoside appears promising. However, further clinical investigation is required.

### 2.3. Alternative Routes of Administration

Promising progress has been made in alternative routes of administration that have increased the bioavailability of orally administrated resveratrol. These are discussed below. 

#### 2.3.1. Inhalation

Dipalmitoylphosphatidylcholine-coated lipid nanoparticles (DPPC-LNs) have been proposed as a potential viable delivery system for site-specific treatment of pulmonary arterial hypertension. Intratracheal administration of RES-loaded DPPC-LNs exhibited an 80% cumulative drug release over a 48-hour period [17]. This is indicative of a longer pulmonary retention of RES, with a slower entry into the systemic blood circulation compared to an intravenously dosed RES solution [17]. Wang et al. [18] demonstrated how the water solubility of RES may be increased 66-fold by aerosolization with a sulfobutylether-β-cyclodextrin (CD-RES) complex loaded onto polymeric nanoparticles [18]. In the in vitro experiments, CD-RES nanoparticles demonstrated improved cellular uptake, cytotoxicity, and apoptosis compared to RES (while antioxidant activity was preserved) [18]. This led to the proposal that CD-RES nanoparticles be used as a potential inhalable delivery system for the treatment of non-small-cell-lung-cancer [18]. Once again, clinical studies are required to validate the efficacy of this approach.

#### 2.3.2. Transdermal

Topical application of RES is not new. However, in recent years many efforts have been made to enhance absorption, aquas stability and UV-dependent stability for the treatment of various skin conditions. Different nanocarriers for topical application hold great potential to enhance RES’s aquas solubility, providing photoprotection and blocking the conversion of the active trans-isomer to an inactive cis-isomer [19,20]. Microemulsions have been found to facilitate superior skin penetration of trans-RES compared to aquas solutions. Additionally, enhanced transdermal bioavailability has been achieved when applying nanostructured emulsions of isopropyl myristate and caproyl 90 as oil phases and the dendrimer-resveratrol complex [19,20,21]. Clinical studies are needed to investigate this further.

#### 2.3.3. Buccal

Resveratrol-loaded mucoadhesive formulations have been proposed for the prevention and treatment of inflammatory conditions of the oral cavity [22]. RES-loaded mucoadhesive tablets were reported to exert a local effect, rather than a systemic one, in a porcine model which was deemed more desirable for local inflammatory conditions such as oral mucositis, lichen planus, erythema multiforme, nicotinic stomatitis, and recurrent aphthous stomatitis [22]. Mucoadhesive cyclodextrin and xanthan gum-based buccal formulations have also been investigated as potential RES delivery systems. RES release was found to be delayed and controlled by diffusion when administered by this route [23]. This buccal delivery system was found promising and was therefore recommended to improve the effectiveness of treatment of various oral diseases (particularly periodontitis) [23].

#### 2.3.4. Nose-to-Brain

Chitosan-coated lipid microparticles loaded with RES showed promise as a direct nose-to-brain delivery system [24]. In an in vivo rat model, nasal administration of the microparticulate carrier system resulted in a marked increase in cerebrospinal fluid bioavailability with no systemic distribution [24]. This direct and specific nose-to-brain delivery system has great potential for neurotherapeutic applications which should be pursued in clinical studies.

### 2.4. Nanotechnology

**Figure 1:**
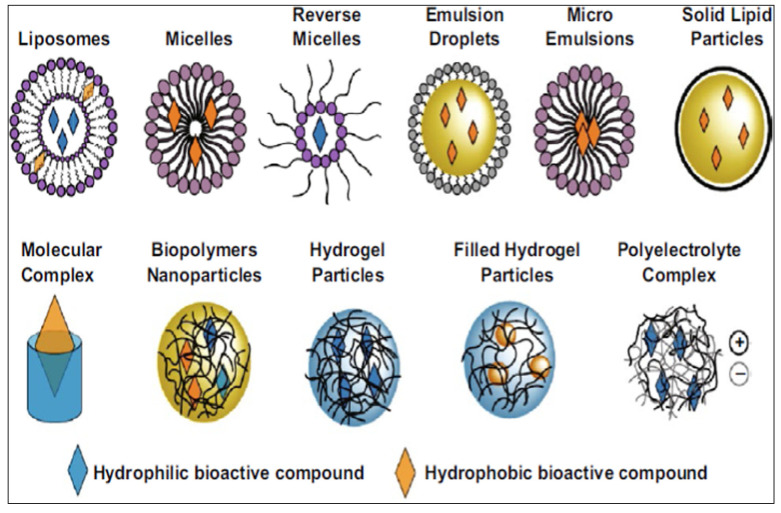
Nano-based delivery systems used to improve the bioavailability of encapsulated pharmaceuticals and nutraceuticals [25,26].

Various nano-based delivery systems have been investigated to improve the bioavailability of encapsulated pharmaceuticals and nutraceuticals, as illustrated in Figure 1 [25,26]. Nanotechnology has been recognized for its promising and superior delivery of natural products for chemoprevention and chemotherapy compared to traditional formulations [2]. The focus on nanotechnology and its various applications in RES delivery systems for potential therapeutic use has expanded since 2017 [27,28,29,30,31]. Lagoa et al. [32] summarized advances in phytochemical delivery systems in an effort to improve anticancer activity. The ability of glyceryl monooleate liquid crystalline nanoparticles to act as a delivery system for RES in urethane-induced lung cancer was determined in mice [33]. Intravenous administration of the loaded nanoparticles resulted in a reduction of tumor mass and malignant surface lesions, the activation of caspase-3, and the inhibition of angiogenesis with greater efficacy than the free drug [33]. Furthermore, the encapsulated formulations improved survival rates and liver and renal safety assessments [33]. 

Zein nanoparticles have been proposed as potentially safe and effective carrier systems to improve the oral bioavailability of RES [34,35]. RES-loaded zein nanoparticles demonstrated low cytotoxicity in human colorectal Caco-2 and HT29-MTX cell lines, with some additional capability to shield RES from metabolism [34]. 

Nanotechnology holds the key to organ targeted therapeutics. Brain targeting was demonstrated by Katekar et al. [36] in an in vivo rat study where trans-resveratrol-loaded mixed micelles were administered intravenously. Although organ targeted therapeutics is regarded as a major advancement, the boundaries have been pushed further to organelle targeted therapy. Following systemic administration of RES-loaded dual-modified novel biometric nanosystems, compounds cross the blood-brain barrier and target neuron cells, specifically by concentrating in the mitochondria [37]. These intravenously administered, neuronal mitochondria-targeted dual-modified novel biomimetic nanosystems may be potential therapeutic candidates for reactive oxygen species-induced mitochondrial dysfunction in Alzheimer’s disease [37]. Organelle targeted nanotherapeutics appear promising and should be studied further.

A review of the potential adverse effects of RES, stresses the need for studies that determine the long-term and adverse effects of RES in humans [38]. Nanotechnology carrier delivery systems are expected to lead to a reduction in the potentially harmful effects of high doses of RES by enhancing its bioavailability, stability, tolerability, safety, and efficacy in humans [38]. For this reason, further research is recommended.

### 2.5. Metabolites 

Due to its rapid metabolism, there has been uncertainty about whether or not RES effects are due to its parent compound or to its active metabolites, which include RES-monosulfate (RES-MS) [39]. Yang et al. determined the metabolic profile of RES in two human bladder cancer (HBC) cell lines, T24 and EJ [29]. Although the T24 cells were more sensitive to RES, both cell lines produced the same metabolite, RES-MS and RES-associated metabolic enzyme, SULT1A1 upregulation was noted [39]. RES showed greater anti-tumor effects than RES-MS and produced a better safety profile in vitro [39]. Although RES was found to be more active than RES-MS in HBC cell lines, studies in other cell lines are recommended.

The intestinal pharmacokinetics of RES and its metabolites, as well as their effect on gut barrier and microbiota was assessed in a CD-1 mouse model [40]. Mice were fasted for 16 h (with access to water ad libitum), after which 50 mg/kg of RES was administered orally. Mice were sacrificed at 0, 0.5, 1, 2, 3, 4, 6, 8, 12, and 24 h after RES ingestion. Then, the gastrointestinal contents were collected and separated into stomach, duodenum, jejunum, ileum, cecum, and colon, and were analysed quantitatively for RES and RES metabolites. Additionally, faeces were collected prior to and at 30 min intervals after RES ingestion. High concentrations of RES and its metabolites were present in the gastrointestinal tract. Although RES and its sulfation metabolites were detected in the intestines and faeces, glucuronidated metabolites were confined to the small intestine [40]. Resveratrol-3-O-sulfate was found to better regulate gut microbial growth and provide superior gut barrier function than RES [40]. This research has provided significant insight into the intestinal metabolism of RES. Further comparative studies to ascertain whether or not RES metabolites are more beneficial than RES would be of value.

### 2.6. Dose-Manipulation

Dose-escalation studies prior to 2018 demonstrated poor results with respect to bioavailability enhancement, with RES displaying linear pharmacokinetics, even at high dosages [2]. No articles matched the set search criteria, implying that no dose-escalation studies have been conducted since the first review. This may be attributed to inter-individual genomic variations in metabolism [41]. In addition, the focus has mainly been on nanotechnology delivery systems, which have indicated more promising results with respect to bioavailability enhancement, safety, efficacy, and RES’s targeted therapeutic potential.

### 2.7. Naturally Occurring Resveratrol Analogues

Various RES analogues, all of which have a more favourable pharmacokinetic profile than RES, have been investigated. These include resveratrol trimethyl ether (trans-3,5,4′-trimethoxystilbene, RTE), pterostilbene (trans-3,5-dimethoxy-4′-hydroxystilbene, PTS), oxyresveratrol (trans-3,5,2′,4′-tetrahydroxystilbene, OXY), isorhapontigenin (trans-3,5,4′trihydroxy-3′-methoxystilbene, ISO), trans-4-4′-dihydrostilbene (DHS), and, more recently, (-)-hopeaphenol and its (dihydro)benzofuran dimers.

Although OXY possesses a better pharmacokinetic profile compared to RES, it is unstable in an aqueous solution and has poor bioavailability. This limitation was addressed by increasing OXY solubility via encapsulation with cyclodextrins [42]. Unfortunately, the release was too rapid, which is a well-known characteristic of cyclodextrins [43]. In an attempt to increase solubility and modulate drug release, OXY was complexed with cycodextrin-based nanosponges [44]. Although the objectives of this strategy were not achieved, encapsulation afforded protection of OXY and resulted in an increase in bioactivity, specifically in anticancer activity [44]. To further enhance its pharmacokinetics, Junsaeng et al. [45] combined OXY with piperine. This complex was administered either intravenously or via oral gavage to male Wistar rats, resulting in a higher OXY plasma concentration as well as a 2-fold increase in oral bioavailability [45]. Furthermore, intravenous administration of the combination resulted in a reduction in OXY glucuronidation as well as an increase in brain tissue concentrations.

Yeo et al. [46] compared the pharmacokinetic profile of ISO to RES, and reported that ISO was approximately 50% more orally bioavailable than RES. Unfortunately, the concentrations of ISO and RES used in these studies were dissimilar. Dai et al. [47] used similar doses of OXY and RES (90 µmol/kg intravenous and 200 µmol/kg orally), and eight additional daily repeated oral doses (100 µmol/kg) in Sprague-Dawley rats. Compared to RES, ISO’s more favourable pharmacokinetic characteristics, long systemic residence, and unaltered exposure after repeated oral dosing was confirmed [47], indicating that ISO is the preferred candidate for drug development and future clinical studies.

(-)-Hopeaphenol, a dihydobenzofuran based resveratrol tetramer, has antibacterial activity [48]. However, due to the complex core structure of this compound (i.e., multiple fused rings and various stereocenters) synthetic synthesis of the compound is challenging. Sundin et al. [49] investigated the inhibitory effects of natural (dihydro)benzofuran RES dimers of T3SS that target Yersinia pseudotuberculosis and Pseudomonas aeruginosa and compared it to (-)-hopeaphenol. The dimers were found to have superior activity compared to the parent compound and were thus recommended as leads for drug discovery and potential clinical development.

## 3. Materials and Methods

A literature search was conducted to extract all articles published after 2017, pertaining to the aspects addressed in a previous review. The databases search included PubMed, Scopus, Science Direct and Google Scholar. Specific keywords included: ‘resveratrol and clinical trials’ and ‘piperine’; ‘3,5,4′tri-O-acetylresveratrol (TARES)’; ‘routes of administration’; ‘nanotechnology’; ‘metabolites’; ‘red grape cells (RGC)’; ‘dose-escalation’; ‘analogues’; ‘Resveratrol trimethyl ether’; ‘Pterostilbene’; ‘Oxyresveratrol’; ‘Isorhapontigenin’; ‘Trans-4-4′-dihydrostilbene’; and ‘resveratrol and synergism’.

## 4. Conclusions

This updated brief review on advances in enhancing the bioavailability of resveratrol showed that RES has continued to demonstrate promising activity, either alone or in combination with other agents, across various therapeutic areas in pre-clinical studies including in cancer, cardiac, lung injury, SARS-CoV-2, and neurodegeneration models. However, clinical efficacy and safety data (as well as dose escalation studies) are still lacking. Although the RES parent compound appears more pharmacologically active than its metabolites, novel approaches to RES administration have increased its suboptimal bioavailability. In addition, various exciting nanotechnology applications that provide targeted RES delivery mechanisms, including to cellular organelles, appear promising. RES analogues may have improved bioavailability, and should be explored as alternatives to RES. Importantly, the need for clinical trials remains paramount in order to validate the clinical efficacy and safety of RES, its metabolites, and various analogues.

## Data Availability

Not applicable.
